# Efficacy and Safety of Immunotherapy with Interferon-Gamma in the Management of Chronic Sulfur Mustard-Induced Cutaneous Complications: Comparison with Topical Betamethasone 1%

**DOI:** 10.1100/2012/285274

**Published:** 2012-03-12

**Authors:** Yunes Panahi, Amirhossein Sahebkar, Seyyed Masoud Davoudi, Mojtaba Amiri, Fatemeh Beiraghdar

**Affiliations:** ^1^Chemical Injuries Research Center, Baqiyatallah University of Medical Sciences, Molla-Sadra Street, Tehran, P.O. Box 19945-581, Tehran, Iran; ^2^Biotechnology Research Center and School of Pharmacy, Mashhad University of Medical Sciences, P.O. Box 91775-1365, Mashhad, Iran; ^3^Nephrology and Urology Research Center, Baqiyatallah University of Medical Sciences, P.O. Box 19945-581, Tehran, Iran

## Abstract

The present trial investigated the efficacy of immunotherapy with interferon-gamma (IFN-*γ*) in the treatment of sulfur mustard (SM)-induced chronic skin complications. Forty subjects who were suffering from chronic skin complications of SM and were diagnosed to have severe atopic dermatitis, were assigned to IFN-*γ* (50 *μ*g/m^2^) subcutaneously three times per week (*n* = 20) or betamethasone valerate topical cream 0.1% (*n* = 20) every night for 30 days. Extent and intensity of cutaneous complications was evaluated using scoring atopic dermatitis (SCORAD) index, and quality of life using dermatology life quality index (DLQI) at baseline and at the end of trial. SCORAD-A and SCORAD-B scores were significantly decreased in both IFN-*γ* and betamethasone. However, SCORAD-C score was decreased only in the IFN-*γ* group. There were significant reductions in overall as well as objective SCORAD scores in both groups. As for the magnitude of changes, treatment with IFN-*γ* was associated with greater reductions in overall, objective and segmented SCORAD scores compared to betamethasone. DLQI reduction was found to be significantly greater in the IFN-*γ* group. Promising improvements in quality life and clinical symptoms that was observed in the present study suggest the application of IFN-*γ* as an effective therapy for the management of SM-induced chronic skin complications.

## 1. Introduction

Sulfur mustard (bis-(2-chloroethyl) sulfide; SM) is an alkylating chemical warfare agent which possesses potent mutagenic, carcinogenic, cytotoxic, and blistering properties [1]. Due to its powerful alkylating nature [2, 3], SM is capable of reacting with multiple vital biomolecules such as lipids, proteins, and DNA, thereby leading to the impairment of several body organs immediately after exposure [4, 5]. Skin is among the primary targets of SM which, due to its high penetrability and high surface area, undergoes the most severe damage [6].

Cutaneous complications of SM are not limited to the acute phase but late complications may be present even 15–20 years post exposure. These chronic complications have been repeatedly reported in Iranian patients and shown to significantly affect the quality of life [7–9]. The severity of cutaneous complications is a function of dose and duration of SM exposure [10].

During Iraq-Iran war (1983–88), SM was used against Iranian veterans and civilians for several times rendering about 100.000 Iranians chemically injured of whom a considerable number are still suffering from chronic complications [7, 8, 11]. Chronic skin complications of SM include pruritus (being the most frequent with an incidence of 70–90%), burning sensation, pain erythema, xerosis, hyper- and hypopigmentation, and scarring, atrophy [8, 12–14]. These chronic complications have been repeatedly reported in Iranian patients even 15–20 years post exposure and shown to significantly affect the quality of life [8, 9, 12–14].

In most occasions, SM-induced chronic skin complications are in the form of atopic dermatitis (AD). AD is a common and chronic inflammatory skin disorder which is characterized by eczematous lesions, erythema, xerosis, and severe pruritus. So far, multiple factors have been identified as etiologies of AD such as allergy, climatic conditions, microbial infections, and epidermal dysfunction. However, immune imbalance is regarded as the major pathomechanism of AD [15]. Impaired cellular immunity, reduced IFN-*γ* and elevated IgE, interleukin-4 (IL-4), and IL-13 are among the frequent immunological features observed in AD. The constellation of these imbalances increases the susceptibility of patient to viral and fungal infections [16].

IFN-*γ*, also known as type II interferon, is a cytokine which is critically involved in the innate as well as adaptive immunity. IFN-*γ* is produced by Th_1_ cells, cytotoxic T cells, and NK cells, and it has antiviral, antibacterial, antiproliferative, and immunomodulatory properties [17]. As a cytokine being markedly decreased in AD, the therapeutic effectiveness of IFN-*γ* in AD has been investigated by a number of trials [18–20]. The results have consistently confirmed the efficacy and safety of IFN-*γ* for AD at both short- and long-term use. However, IFN-*γ* has not yet been tested in the alleviation of SM-induced AD symptoms. Current strategies for the control of SM-induced chronic skin complications are mainly symptomatic and limited to topical corticosteroids, antihistamines, and local moisturizers. Regarding the significant impact of these complications—in particular pruritus—on patients' daily functioning, sleeping, and quality of life [21], there is an urgent demand for safe and effective drugs to be used against SM-induced AD [22, 23]. To this end, the present trial sought to investigate for the first time the efficacy and safety of immunotherapy with IFN-*γ* in the treatment of SM-induced chronic skin complications.

## 2. Methods

### 2.1. Participants

Included subjects were chemically injured patients who were suffering from chronic skin complications of SM and were diagnosed to have severe AD. Patients were excluded if they had a SCORAD score <50, received IFN-*γ* before the initiation of trial, or had history of diabetes mellitus, coagulopathy, myelosuppression, or any psychological, central nervous system, hepatic, renal, cardiovascular, thyroid, pulmonary, and autoimmune disorder. These subjects were exposed to SM some 20 years ago and are registered in our center, have medical histories and documents, and periodically visited in the center. Based on previous documents, dermatologic examination in the present trial, and their self report in the study questionnaire, these subjects did not have a history of AD, vitiligo, pemphigus, psoriasis, and contact dermatitis. Regarding the known potent dermatotoxic effects of sulfur mustard on the skin, it is evident that the cutaneous complications of these subjects are secondary to the mustard exposure.

### 2.2. Design

This investigation was a randomized clinical trial which was performed in the Baqiyatallah Hospital (Tehran, Iran). Forty patients were recruited into the trial. These patients were randomly assigned to receive IFN-*γ* (50 *μ*g/m^2^) subcutaneously three times per week (IFN-*γ* group; *n* = 20) or betamethasone valerate topical cream 0.1% (betamethasone group; *n* = 20) every night for 30 days. Patients were instructed by the study nurse to perform IFN-*γ* injections by self and at bedtime. Topical betamethasone was recommended to be applied on affected skin area(s) one hour before bedtime. They were also recommended to avoid contact with allergens and other disease intensifying factors and use acetaminophen in case of flu-like symptoms.

The study protocol was approved by the Ethics Committee of the relevant University and written informed consent was obtained from participants.

### 2.3. Efficacy Measures

 Extent and intensity of cutaneous complications was evaluated using scoring atopic dermatitis (SCORAD) index. This index was developed by the European Task Force on Atopic Dermatitis (ETFAD) in 1993 based on a broad consensus by dermatologists [24].

SCORAD index consists of three parts: SCORAD-A: extent of the disorder which is calculated based on the rule of nines; SCORAD-B: intensity of the disorder which comprises assessment and grading of six items as follows: erythema, oedema/papulation, excoriations, lichenification, oozing/crusts and dryness; and SCORAD-C: subjective symptoms including pruritus and sleeplessness. Total SCORAD score is calculated using the formula *A*/5 + 7*B*/2 + *C*. The maximum scores of parts *A*,* B*, and *C* are 100, 18, and 20, respectively. Therefore, the SCORAD index would range between 0 and 103. Objective SCORAD is a modified form of SCORAD index in which the subjective part—that appeared to be a source of large variations—has been eliminated and the concentration is focused on objective signs (extent and intensity). The range of objective SCORAD lies between 0 and 83. Based on the Overall SCORAD and objective SCORAD, the severity of disease could be classified into mild (<15 for objective and <25 for overall SCORAD), moderate (15–40 for objective and 25–50 for overall SCORAD), or severe (≥40 for objective and ≥50 for overall SCORAD). In the present study, overall and segmented SCORAD scores were determined at baseline and at the end of trial.

### 2.4. Biochemical Measurements

A complete blood count together with serum levels of Na, K, creatinine, blood urea nitrogen (BUN), aspartate aminotransferase (AST), alanine aminotransferase (ALT), and alkaline phosphatase (ALKP) was determined for patients using routine laboratory methods at baseline and after 4 weeks of treatment. In addition, baseline and post trial serum concentrations of IL-4, IL-6, IgE, and IFN-*γ* were determined in the IFN-*γ* group to assess the impact of immunotherapy on these measures.

### 2.5. Quality of Life Assessment

Dermatology Life Quality Index (DLQI) was employed to evaluate the impact of IFN-*γ* and topical betamethasone on patients' quality of life. DLQI is a simple, practical, and widely used index for the assessment of health-related quality of life and has been previously employed in Iranian population including subjects with SM-induced chronic skin lesions [21]. In addition, the validity and reliability of this index has been confirmed in an Iranian population [25]. DLQI questionnaire consists of 10 questions under 6 headings: symptoms and feelings (questions 1 and 2), daily activities (questions 3 and 4), leisure (questions 5 and 6), work and school (question 7), personal relationships (questions 8 and 9), and treatment (question 10). Each question has a maximum score of 3 and options of “very much” (scored 3), “a lot” (scored 2), “a little” (scored 1), and “not at all” (scored 0). In addition, a “0” score is allotted to “not relevant” response and unanswered questions. DLQI total score is calculated by summing the scores of all questions, resulting in a scoring range of 0–30 in which higher scores are associated with more severe impairments of life quality. DLQI scores of 0-1, 2–5, 6–10, 11–20, and 21–31 are equivalent to no, small, moderate, very large, and extremely large effects on patient's quality of life, respectively.

### 2.6. Statistical Analyses

Statistical analyses were performed using SPSS software for Windows (version 16). Data were expressed as mean ± SEM or number (%). Between and within groups comparisons were made using independent samples *t*-test and paired samples *t*-test, respectively. Categorical variables were compared using Fisher's Exact test. Bivariate correlations between different parameters were assessed using Pearson's correlation coefficients. A two-sided *P* value of <0.05 was considered to be statistically significant.

## 3. Results

**Figure 1:**
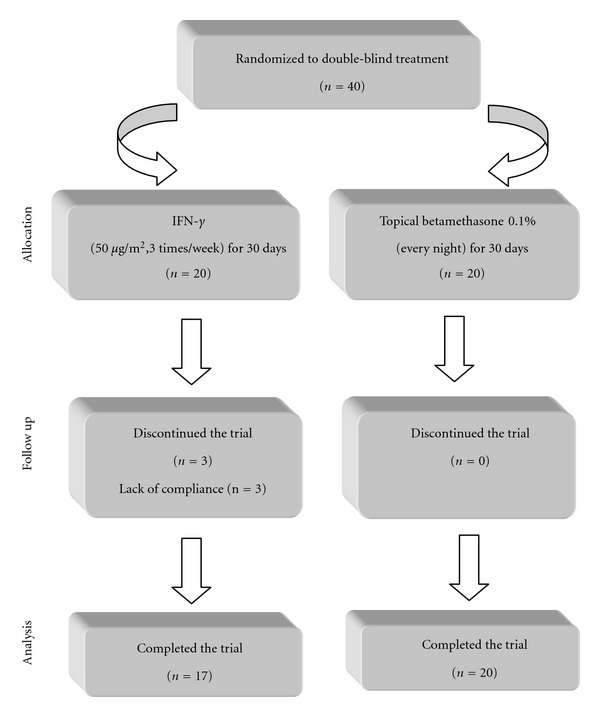
Flowchart of the trial.

From the 40 subjects who were initially entered the trial, only 3 were dropped out of study in the betamethasone group due to lack of compliance. Therefore, 37 subjects completed the trial (*n* = 20 and 17 in the IFN-*γ* and betamethasone groups, resp.; Figure 1) and were included in the final analyses. Drop-out rate was not significantly different between the groups (*P* > 0.05).

### 3.1. Demographic Characteristics

The groups were comparable regarding their age (38.90 ± 2.45 versus 39.12 ± 3.30 for IFN-*γ* and betamethasone group, resp.) and demographic findings including weight, height, and BMI at baseline. Likewise, there was no significant difference between IFN-*γ* and betamethasone groups in their vital signs including pulse rate, respiratory rate, body temperature, and systolic and diastolic blood pressure (*P* > 0.05). Family histories of allergic rhinitis (15.0% versus 0%) and atopic dermatitis (15.8% versus 0%) were also comparable between the groups (*P* > 0.05).

### 3.2. SCORAD Index

As referred previously, extent, intensity, and subjective symptoms of atopic dermatitis are reflected in parts *A*, *B*, and *C* of the SCORAD index, respectively. SCORAD-*A* and SCORAD-*B* scores were significantly decreased by the end of trial in both IFN-*γ* (*P* < 0.001 for both SCORAD-*A* and SCORAD-*B*) and betamethasone (*P* = 0.004 for SCORAD-*A* and *P* = 0.044 for SCORAD-*B*). However, SCORAD-*C* score was decreased only in the IFN-*γ* group (*P* < 0.001) while no significant change was observed in the betamethasone group (*P* > 0.05). There were significant reductions in overall SCORAD scores in both groups (*P* < 0.001 in the IFN-*γ* and *P* = 0.006 in the betamethasone group). Objective SCORAD scores were also significantly decreased in both groups by the end of the trial (*P* < 0.001 in the IFN-*γ* and *P* = 0.006 in the betamethasone group) (Table 1).

**Figure 2:**
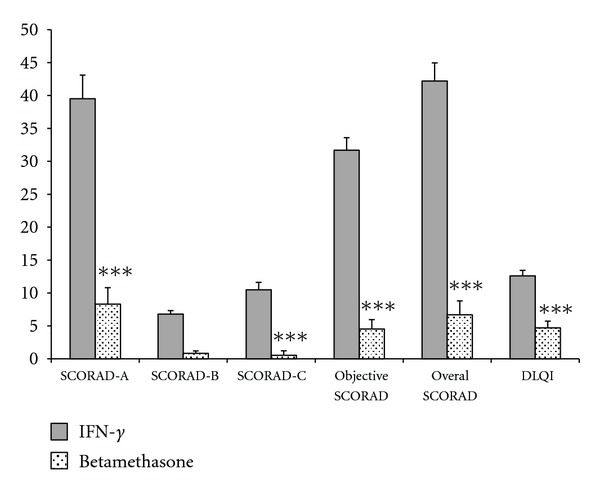
Comparison of the magnitude of changes in SCORAD and DLQI scores between IFN-*γ* and betamethasone groups. ***P < 0.001.

As for the magnitude of changes, treatment with IFN-*γ* was associated with greater reductions in overall (*P* < 0.001), objective (*P* < 0.001), and segmented (*P* < 0.001) SCORAD scores compared to betamethasone (Figure 2). At baseline, both groups had severe AD based on their SCORAD scores. At the end of trial, mean overall SCORAD score was regressed from severe to mild in the IFN-*γ* group. However, in spite of significant reduction, mean overall SCORAD score did not regress to a less severe category in the betamethasone group.

### 3.3. Quality of Life Index

DLQI scores were significantly decreased in both IFN-*γ* (*P* < 0.001) and betamethasone (*P* < 0.001) groups by the end of trial, indicative of the improvement of life quality. As for the magnitude of changes in DLQI scores, no significant difference was observed between the groups (*P* > 0.05) (Table 1).

### 3.4. Biochemical Parameters

Complete biochemistry profile was determined in the collected blood samples. No significant difference was found in the baseline as well as post-trial levels of the assessed parameters, including serum Na, K, AST, ALT, ALKP, Creatinine, BUN, CBC, and total and direct billirubin, between the groups (*P* > 0.05). Likewise, none of these parameters were significantly changed by the end of trial in any of the groups (*P* > 0.05).

Administration of IFN-*γ*, 50 *μ*g/m^2^ three times weekly, was associated with a significant decrease in serum IgE (28.93 ± 0.83 (baseline) versus 22.26 ± 0.75 (post-trial) at baseline and at the end of trial, resp.; *P* < 0.001) and IL-4 (64.21 ± 1.20 (baseline) versus 57.53 ± 1.34 (post-trial); *P* < 0.001) concentrations. In contrast no significant change was observed for serum IL-6 (0.61 ± 0.03 (baseline) versus 0.67 ± 0.05 (post-trial); *P* > 0.05). Administration of IFN-*γ* significantly elevated mean serum IFN-*γ* by the end of trial (41.23 ± 1.19 (baseline) versus 46.57 ± 1.49 (post-trial); *P* = 0.013).

### 3.5. Adverse Events

**Figure 3:**
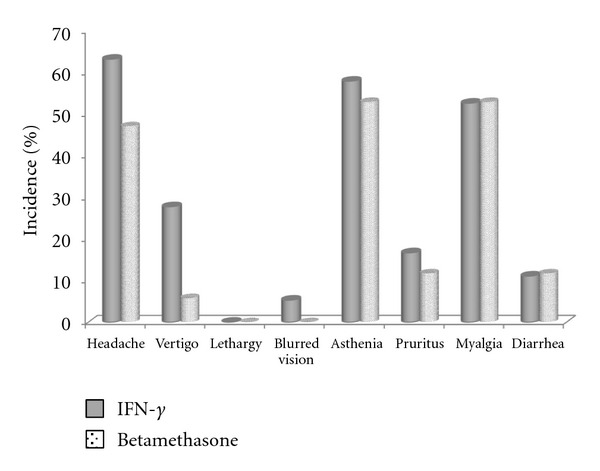
Frequency of evaluated adverse events in the study groups.

**Figure 4:**
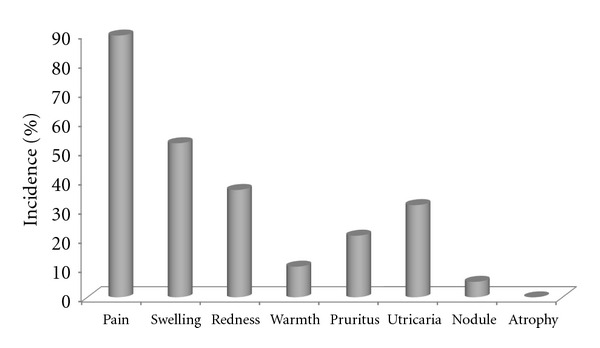
Frequency of injection site adverse events in the IFN-*γ* group.

Headache, vertigo, lethargy, blurred vision, pruritus, myalgia, asthenia, and diarrhea were the adverse effects that were asked from both IFN-*γ* and betamethasone groups. Interestingly, the incidence of none of these adverse events did significantly differ between the groups (*P* > 0.05) (Figure 3). Aside from the aforementioned side effects, the incidence of pain, swelling, redness, warmth, itching, atrophy, utricaria, and nodule formation at the injection site was specifically evaluated in the IFN-*γ* group as summarized in Figure 4.

### 3.6. Bivariate Analysis

Bivariate correlations were assessed between baseline and post-trial values of the efficacy measures (DLQI, SCORAD-*A*, SCORAD-*B*, and SCORAD-*C*) and serum concentrations of IL-4, IL-6, and IgE. Significantly positive correlations were found between baseline DLQI and IL-6 (*P* = 0.01), baseline SCORAD-*B* and IL-4 (*P* = 0.001), and baseline SCORAD-*C* and IL-4 (*P* = 0.001). On the other hand, significantly negative correlations were observed between baseline DLQI and IL-4 (*P* = 0.04), post-trial DLQI and IL-6 (*P* = 0.02), and baseline SCORAD-*B* and IL-6 (*P* = 0.01) (Table 2).

## 4. Discussion

 The present study was designed to determine the effect of IFN-*γ* therapy on the quality of life and severity of symptoms in patients suffering from chronic SM-induced AD. The secondary objective was to compare the efficacy of IFN-*γ* with that of topical betamethasone as the most commonly administered medication for chronic SM-induced cutaneous complications. The results indicated significant improvements in segmented and overall SCORAD as well as DLQI scores in both IFN-*γ* and betamethasone groups. Of note, IFN-*γ* was found to be associated with greater improvement in the evaluated efficacy measures and well tolerated in patients.

SM-induced dermatitis is accompanied by severe and chronic pruritus in most cases. These pruritic symptoms have been shown to affect patients' quality of life [21]. As mentioned previously, chronic cutaneous complications of SM intoxication could be categorized as a form of atopic dermatitis. Unfortunately, no satisfactory treatment has yet been introduced for AD. Although corticosteroids appear as an effective therapeutic approach, their continuous and long-term application is associated with the incidence of several side effects [26], which may lead to the discontinuation of drug. Application of other treatments such as cyclosporine is also limited for the same reason [27].

Immune system is among the multiple organs that are affected by SM. Long-term SM-induced immunological disturbances have been reported in intoxicated patients even 16–20 years post exposure [6, 28]. Severe pruritus and resulting sleeping disorders were among the most important complaints of patients at baseline. These complications, assessed using SCORAD and DLQI indices, were improved in both IFN-*γ* and betamethasone groups but the impact of IFN-*γ* was significantly superior to that of betamethasone. The improvements in SCORAD index and quality of life that were observed in the IFN-*γ* group were in line with the significant reductions in serum levels of IgE and IL-4. Therefore, it is plausible that the beneficial effects of IFN-*γ* are, at least in part, secondary to its well-known immunomodulatory activities [29].

Treatment with IFN-*γ* has been previously shown to be efficacious in the alleviation of AD complications. However, no study has to date evaluated the efficacy of IFN-*γ* in the treatment of SM-induced AD. In a previous survey by Nuttall and colleagues [30], canine atopic dermatitis was associated with overexpression of IL-4 mRNA and reduced transcription of TGF-*β* compared to healthy skin. In addition, significantly higher levels of IFN-*γ*, TNF-*α*, and IL-2 mRNA were seen in lesional atopic compared to nonlesional atopic and healthy skin. In another investigation by Reinhold et al. [18], treatment with IFN-*γ* for 6 weeks caused marked clinical improvement in 8 out of the total of 14 treated patients. Hanifin et al. [19] have reported that treatment with IFN-*γ* for 12 weeks was safe and effective in reducing inflammation, clinical symptoms, and eosinophilia in severe atopic dermatitis. In another comprehensive study by Stevens and colleagues [20], long-term effects of IFN-*γ* therapy were assessed in patients with atopic dermatitis for a period of 2 years. The results indicated that long-term administration of IFN-*γ* is associated with significant reductions in disease extent and clinical severity scores for pruritus, erythema, edema, excoriations, dryness, lichenification, and other atopic symptoms. The efficacy of 4-week recombinant IFN-*γ* has also been evaluated against topical diphenhydramine in the treatment of canine AD [31]. The findings were indicative of the significantly higher efficacy of IFN-*γ* compared to diphenhydramine in the improvement of pruritus, erythema, excoriation, and alopecia.

Aside from efficacy, another important factor that should be considered for an ideal AD therapy is safety issue. Interestingly, the incidence of none of the evaluated side effects did significantly differ between the groups. This is especially important when considering that the comparison was between a parenteral and a topically administered drug. As for the injection site reactions, the incidence of side effects was generally low except for pain and swelling. However, none of the side effects at the site of injection led to the discontinuation of IFN-*γ*. In addition, no adverse effect was observed from IFN-*γ* on blood biochemical parameters including markers of hematological, renal, and hepatic function. These results are consistent with those obtained by Stevens et al., who indicated that IFN-*γ* is well tolerated over a long-term (2 years) period [20].

Along with the disturbances in the immune system, sulfur mustard induces barrier damage through its various apoptotic, necrotizing, and inflammatory effects that all impair skin permeability, connectivity, and biophysical properties. In addition, sulfur mustard alkylates extracellular matrix proteins, thereby impairing the interaction of basal keratinocytes to matrix proteins and their adherence to basement membrane [3, 32–34]. These effects of sulfur mustard are particularly important in stratum corneum which is the main barrier against water loss. In a recent study, transepidermal water loss has been shown to be higher in patients with chronic mustard-induced skin lesions compared to healthy subjects [35]. On the other hand, the same patients have been frequently reported to complain from xerosis (a consequence of damage to the skin's hydrolipidic barrier) [8, 12–14]. Therefore, more research is needed to better understand the impact of immunotherapy with IFN-*γ* on the barrier function of skin in SM-intoxicated patients.

To sum up, the present research provided the first evidence on the efficacy of IFN-*γ* therapy in SM-induced AD. Promising improvements in quality life and clinical symptoms that were observed in the present study suggest the application of IFN-*γ* as an effective therapy for the management of SM-induced chronic skin complications. Since the present trial was not blinded, it would be helpful to confirm the effectiveness of IFN-*γ* in a future double-blind study. It is also recommended that further research be undertaken to elucidate the impact of immunotherapy with IFN-*γ* on other immune and inflammatory biomarkers which are altered in AD. Finally, future trials are warranted to assess the efficacy of IFN-*γ* against other chronic complications of SM.

## Figures and Tables

**Table 1:** Effects of IFN-*γ* versus betamethasone 0.1% on the evaluated efficacy measures.

	IFN-*γ*		*P* value	Betamethasone 0.1%		*P* value
	Pretrial	Posttrial		Pretrial	Posttrial	
DLQI	20.80 ± 0.88	8.20 ± 0.48	<0.001	21.88 ± 0.77	17.18 ± 1.28	<0.001
SCORAD-*A *	68.58 ± 4.47	29.05 ± 3.67	<0.001	44.15 ± 2.07	35.82 ± 2.35	0.004
SCORAD-*B *	11.35 ± 0.33	4.55 ± 0.34	<0.001	9.12 ± 0.57	8.29 ± 0.59	0.044
SCORAD-*C *	16.60 ± 1.03	6.10 ± 0.70	<0.001	15.94 ± 0.65	15.41 ± 0.84	>0.05
Objective SCORAD	53.44 ± 1.16	21.73 ± 1.77	<0.001	40.74 ± 1.89	36.19 ± 2.01	0.006
Overall SCORAD	71.44 ± 2.45	23.95 ± 1.78	<0.001	58.31 ± 1.88	51.60 ± 2.75	0.006

Values are expressed as mean ± SEM. DLQI: dermatology life quality index; SCORAD: scoring atopic dermatitis. SCORAD-*A*, -*B*, and -*C* denote extent, intensity, and subjective symptoms of the disorder, respectively.

**Table 2:** Bivariate analyses in the IFN-*γ* group.

		IL-4		IL- 6		IgE	
		*r*	*P* value	*r*	*P* value	*r*	*P* value
DLQI	Pretrial	**−0.48**	**0.04**	**0.61**	**0.01**	−0.27	0.28
	Post-trial	−0.06	0.81	**−0.54**	**0.02**	0.02	0.92
SCORAD-*A *	Pretrial	−0.18	0.48	**0.47**	**0.05**	−0.03	0.9
	Post-trial	−0.24	0.34	0.13	0.61	−0.29	0.24
SCORAD-*B *	Pretrial	**0.88**	**0.001**	**−0.56**	**0.01**	0.32	0.2
	Post-trial	−0.06	0.81	0.24	0.35	−0.22	0.39
SCORAD-*C *	Pretrial	**0.74**	**0.01**	−0.23	0.35	0.13	0.61
	Post-trial	−0.12	0.63	0.04	0.87	−0.14	0.57

DLQI: dermatology life quality index; SCORAD: scoring atopic dermatitis. SCORAD-*A*, -*B*, and -*C* denote extent, intensity, and subjective symptoms of the disorder, respectively.
